# From Normal Cognition to Cognitive Impairment and Dementia: Impact of Orthostatic Hypotension

**DOI:** 10.1161/HYPERTENSIONAHA.121.17454

**Published:** 2021-07-06

**Authors:** Xin Xia, Rui Wang, Davide L. Vetrano, Giulia Grande, Erika J. Laukka, Mozhu Ding, Laura Fratiglioni, Chengxuan Qiu

**Affiliations:** 1 Aging Research Center, Department of Neurobiology, Care Sciences and Society (NVS), Karolinska Institutet-Stockholm University, Sweden (X.X., R.W., D.L.V., G.G., E.J.L., M.D., L.F., C.Q.).; 2 The Swedish School of Sport and Health Sciences, GIH, Stockholm, Sweden (R.W.).; 3 Department of Medicine and Wisconsin Alzheimer’s Disease Research Center, University of Wisconsin School of Medicine and Public Health, Madison (R.W.).; 4 Department of Geriatrics, Catholic University of Rome, Italy (D.L.V.).; 5 Centro di Medicina dell’Invecchiamento, Fondazione Policlinico A. Gemelli, Rome, Italy (D.L.V.).; 6 Stockholm Gerontology Research Center, Sweden (E.J.L., L.F.).; 7 Unit of Epidemiology, Institute of Environmental Medicine, Karolinska Institutet, Stockholm, Sweden (M.D.).

**Keywords:** cohort studies, dementia, hypotension, orthostatic, population

## Abstract

The role of orthostatic hypotension (OH) in the continuum of cognitive aging remains to be clarified. We sought to investigate the associations of OH with dementia, cognitive impairment, no dementia (CIND), and CIND progression to dementia in older adults while considering orthostatic symptoms. This population-based cohort study included 2532 baseline (2001–2004) dementia-free participants (age ≥60 years; 62.6% women) in the SNAC-K (Swedish National Study on Aging and Care in Kungsholmen) who were regularly examined over 12 years. We further divided the participants into a baseline CIND-free cohort and a CIND cohort. OH was defined as a decrease by ≥20/10 mm Hg in systolic/diastolic blood pressure upon standing and further divided into asymptomatic and symptomatic OH. Dementia was diagnosed following the international criteria. CIND was defined as scoring ≥1.5 SDs below age group-specific means in ≥1 cognitive domain. Data were analyzed with flexible parametric survival models, controlling for confounding factors. Of the 2532 participants, 615 were defined with OH at baseline, and 322 were diagnosed with dementia during the entire follow-up period. OH was associated with an adjusted hazard ratio of 1.40 for dementia (95% CI, 1.10–1.76), 1.15 (0.94–1.40) for CIND, and 1.54 (1.05–2.25) for CIND progression to dementia. The associations of dementia and CIND progression to dementia with asymptomatic OH were similar to overall OH, whereas symptomatic OH was only associated with CIND progression to dementia. Our study suggests that OH, even asymptomatic OH, is associated with increased risk of dementia and accelerated progression from CIND to dementia in older adults.

## Abstract 2

Supplemental Digital Content is available in the text.

Orthostatic hypotension (OH) is a common condition in older adults and reflects an insufficient hemodynamic adaptation to postural changes.^1^ OH can be generally divided into neurogenic OH caused by neurodegenerative disorders (eg, Parkinson disease) and non-neurogenic OH caused by other factors such as using medications that can decrease the cardiac sympathetic tone and conditions that can lead to reduced blood volume.^1^ A few population-based studies in middle-aged and older people have linked OH with an elevated risk of dementia, and cerebral hypoperfusion may partly explain the association.^2–6^ However, most people with OH do not report any symptoms linked to cerebral hypoperfusion, indicating large interindividual variability in cerebrovascular autoregulation.^7–9^ Notably, it remains to be clarified whether the association of OH with dementia varies by the presence or absence of cerebral hypoperfusion-related orthostatic symptoms. Besides, the role of OH in the prodromal dementia phases is poorly understood. Data from a memory clinic setting showed a higher prevalence of OH in people with cognitive impairment than those with normal cognition,^10^ but evidence from longitudinal studies is mixed.^2,5,11–18^ Furthermore, OH has been associated with the progression from cognitive impairment to dementia in a memory clinic setting,^19^ whereas evidence from population-based studies is lacking.

This population-based cohort study of Swedish older adults aimed to increase the understanding of OH’s role in the cognitive continuum from normal cognitive function to cognitive impairment and dementia. Specifically, we sought to (1) examine the association of OH with dementia, (2) evaluate the association of OH with cognitive impairment, no dementia (CIND), and (3) assess the association of OH with the progression from CIND to dementia. We also considered orthostatic symptoms when examining these associations.

## Methods

This study used data from the ongoing SNAC-K (Swedish National Study on Aging and Care in Kungsholmen). The anonymized data from the SNAC-K study are accessible when the administrative committee of the SNAC-K study at the Aging Research Center, Karolinska Institutet, Stockholm, Sweden, approves the request (www.snac-k.se).

### Study Design and Participants

This is a population-based cohort study using data from the SNAC-K, a multidisciplinary study of aging and health among people aged ≥60 years, living at home or in institutions in the Kungsholmen area of central Stockholm.^20^ The study population consists of samples randomly selected from 11 age groups (60, 66, 72, 78, 81, 84, 87, 90, 93, 96, and 99+ years). Between 2001 and 2004, 3363 (73.3%) of the 4590 invited individuals participated in the baseline examination. After that, the younger cohorts (age <78 years) are examined every 6 years, and the older cohorts (age ≥78 years) are examined every 3 years. All waves of examination in the SNAC-K study and the linkage of SNAC-K data with register data were approved by the Regional Ethical Review Board in Stockholm, Sweden. Written informed consent was obtained from all participants or a proxy if the participant was cognitively impaired.

**Figure 1.**
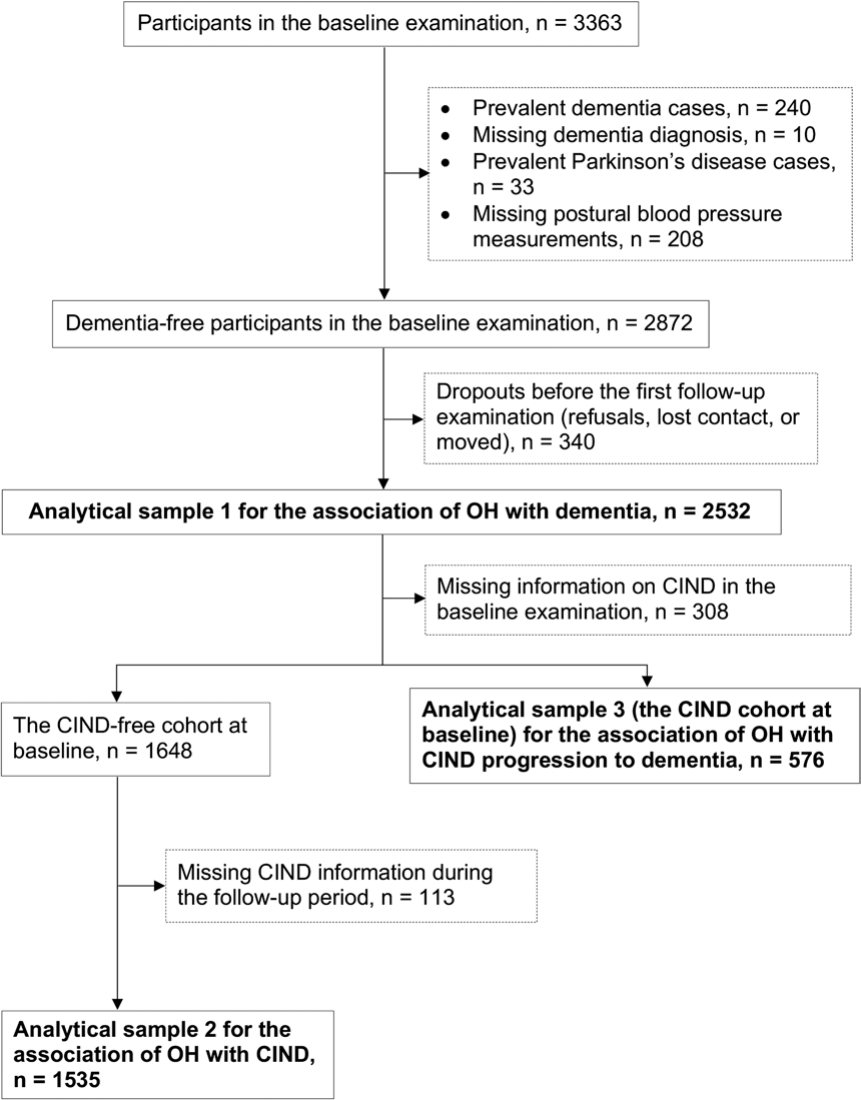
**Flowchart of the study participants.** CIND indicates cognitive impairment, no dementia; and OH, orthostatic hypotension.

Our study used data from 2001 to 2004 (baseline) to 2013 to 2016 and defined 3 analytical samples to address the 3 specific research objectives (Figure 1). To evaluate the OH-dementia association, we excluded individuals with dementia (n=240), missing dementia diagnosis (n=10), with Parkinson disease (n=33), or missing postural blood pressure (BP) measurements (n=208) at baseline from the 3363 participants. Of the 2872 (85.4%) dementia-free people, 340 (11.8%) individuals were further excluded due to dropout, leaving 2532 persons in the dementia-free cohort (analytical sample 1). Of these, 2224 (87.8%) persons had information to define CIND at baseline and were divided into a baseline CIND-free cohort (n=1648) and a CIND cohort (n=576). For the OH-CIND association, we used data from the baseline CIND-free cohort and further excluded 113 (6.9%) people due to lack of information to define CIND during the follow-up period, leaving 1535 participants in analytical sample 2. We used data of the CIND cohort (analytical sample 3) to analyze the association between baseline OH and the progression from CIND to dementia.

### Ascertainment of OH

In the baseline examination, BP was measured 4 times from the left arm with a sphygmomanometer in a quiet room with a constant temperature.^21^ BP in the sitting position was measured twice; at each time, BP was measured after a 5-minute rest. The third BP reading was measured after a 5-minute rest in the supine position. Then, participants were instructed to stand up, and the fourth BP reading was measured after 1 minute of standing. OH was defined as a drop of ≥20 mm Hg in systolic BP (SBP) or ≥10 mm Hg in diastolic BP (DBP) from the third to fourth reading.^1^ Symptoms upon 1 minute of standing were explicitly asked and recorded. We considered light-headedness, dizziness, fatigue, weakness, syncope, visual disturbances, and hearing disturbances as orthostatic symptoms related to cerebral hypoperfusion.^22^

### Diagnosis of Dementia and Alzheimer Disease

At each study visit, all participants were examined via structured interviews (eg, lifestyle factors and health history) and a clinical examination (eg, chronic health conditions, use of medications, and physical functioning). Cognitive tests used for dementia diagnosis included the Mini-Metal State Examination, the clock drawing test, and the Digit Span Forward and Backward tests. Dementia was diagnosed according to the Diagnostic and Statistical Manual of Mental Disorders, fourth edition criteria following a 3-step procedure.^23^ In brief, the examining physician made the first diagnosis based on interviews and clinical examinations. Then, another physician made the second diagnosis independently by reviewing all records from interviews and clinical examinations. A neurologist external to the data collection made a final diagnosis in case of disagreement between the first and second diagnoses. The National Institute of Neurological and Communicative Disorders and Stroke and the Alzheimer Disease and Related Disorders Association criteria were applied for the diagnosis of Alzheimer disease (AD).^24^ We did not analyze other types of dementia separately due to too few cases. Among the deceased participants who had not been diagnosed with dementia, dementia cases were identified through medical charts reviewed by physicians in the SNAC-K research group and through linkage to the Swedish Cause of Death Register.

### Diagnosis of CIND

Trained psychologists assessed cognitive function during each wave of examination using a comprehensive neuropsychological battery involving 5 cognitive domains: episodic memory (free recall), executive function (trail making test, part B), language (category and letter fluency), visuospatial abilities (mental rotations), and perceptual speed (digit cancellation and pattern comparison).^25,26^ The raw score for each test was standardized into a *Z* score using the baseline mean and SD, and the average of *Z* scores was generated for a specific domain when more than one test was performed.^26^ CIND was defined as a test score of ≥1.5 SDs below the age group-specific mean in at least one cognitive domain, without meeting the criteria for dementia.^26^

### Assessments of Covariates

Education was dichotomized as below high school and high school or above. Body mass index was categorized as normal (<25 kg/m^2^), overweight (25–29.9 kg/m^2^), and obesity (≥30 kg/m^2^). Smoking was dichotomized as never and ever smoking. Heavy alcohol drinking was defined as consuming ≥8 drinks/wk for women and ≥15 drinks/wk for men. Physical inactivity was defined as light/moderate/intensive exercise ≤2 to 3 times per month. Diabetes was determined through the following sources: glycated hemoglobin (HbA1c ≥6.5%), a record of diabetes in the Swedish National Patient Register, self-reported history of diabetes, and use of oral blood glucose-lowering medication or insulin injection. High cholesterol level was defined as having nonfasting total serum cholesterol ≥6.22 mmol/L or current use of lipid-lowering drugs. Antihypertensive medication included diuretics, α-blockers, β-blockers, β-/α-blockers, angiotensin-converting enzyme inhibitors, angiotensin II receptor blockers, and calcium channel blockers and other vasodilators. Walking speed was used as a measure of motor function and measured through a 2.4- or 6-meter walk test; slow walking speed was defined as <0.8 m/s. Heart diseases, including atrial fibrillation, heart failure, and ischemic heart disease, were ascertained by integrating information from the baseline interviews, clinical examination, and the Swedish National Patient Register^27^ and categorized as having 0, 1, and ≥2 heart diseases. Cerebrovascular disease was ascertained through the same procedure.^27^ Genotyping of *APOE* was performed using matrix-assisted laser desorption/ionization time-of-flight analysis on a modified Sequenom MassARRAY platform at the Mutation Analysis Facility, Karolinska Institutet, and *APOE* genotype was divided as carrying any ε4 allele versus no ε4 allele.

### Statistical Analysis

Baseline characteristics of study participants by OH status were compared using logistic regression and linear regression models adjusted for age.

We used flexible parametric survival models to estimate the hazard ratio and 95% CI of dementia and AD associated with baseline OH using follow-up time as the time scale. We further divided OH into asymptomatic and symptomatic OH in the models. To evaluate the associations of OH with cognitive outcomes by the severity of OH, we also divided OH into mild OH (SBP drops by 20–29 and DBP drops by 10–14 mm Hg after 1 minute of standing) and severe OH (SBP/DBP drops ≥30/15 mm Hg after 1 minute of standing).^3^ The time of dementia onset was defined as the date of study visit on which the participants were first diagnosed with dementia or the date of death for dementia cases identified from medical charts and the Swedish Cause of Death Registry. The follow-up time was defined as the time interval between the date of the baseline examination and the date of dementia onset, death, or end of follow-up, whichever came first. We first adjusted for age, sex, and education in the models, then additionally adjusted for body mass index, smoking, heavy alcohol drinking, physical inactivity, diabetes, high cholesterol level, SBP in the sitting position (continuous), antihypertensive medication use, slow walking speed, number of heart diseases, and cerebrovascular disease. We applied the same analytical procedures for the associations of OH with incident CIND and CIND progression to dementia or AD, except that for the OH-CIND association, the follow-up time was defined as the time interval between the date of the baseline examination and the date of CIND onset, death, or the time when the individuals were censored due to lack of subsequent CIND information, whichever came first. We also explored the interaction of OH with sex and *APOE* ε4 allele on the risk of the cognitive outcomes by including the individual variables and their interaction terms simultaneously in the models. Finally, we estimated the standardized cumulative incidence of dementia by OH status using flexible parametric survival models while considering the competing risk of death.^28^

We performed the following sensitivity analyses by (1) applying a more stringent definition of OH (SBP/DBP declines ≥30/15 mm Hg after 1 minute of standing) among people with supine hypertension (SBP/DBP ≥150/90 mm Hg in the supine position)^29^; (2) dividing OH into non-neurogenic and neurogenic OH (defined as the ratio of the heart rate increase to SBP decline <0.5 bpm/mm Hg when standing up from the supine position) to explore if the associations of OH with cognitive outcomes differ by OH cause^30^; and (3) exploring the impact of potential reverse causality on the OH-dementia associations by setting a lag period of 3 years between OH assessment and dementia assessment in the survival models.

We dealt with missingness in covariates with multiple imputation by chained equations. Statistical analyses were performed with R software version 3.6.2 (R Foundation for Statistical Computing, Vienna, Austria) and Stata Statistical Software: Release 16.0 (StataCorp, College Station, TX).

## Results

The mean age of the 2532 participants in the dementia-free cohort was 73.1 (SD, 10.4) years, 62.6% were women, and 615 (24.3%) had OH (545 without and 70 with symptoms; 434 with mild OH and 181 with severe OH) at baseline (Table 1). Compared with people without OH, those with OH were older, less obese, more likely to have diabetes, have lower SBP in the sitting position, and more likely to have slow walking speed and ≥2 heart diseases (Table 1).

**Table 1.**
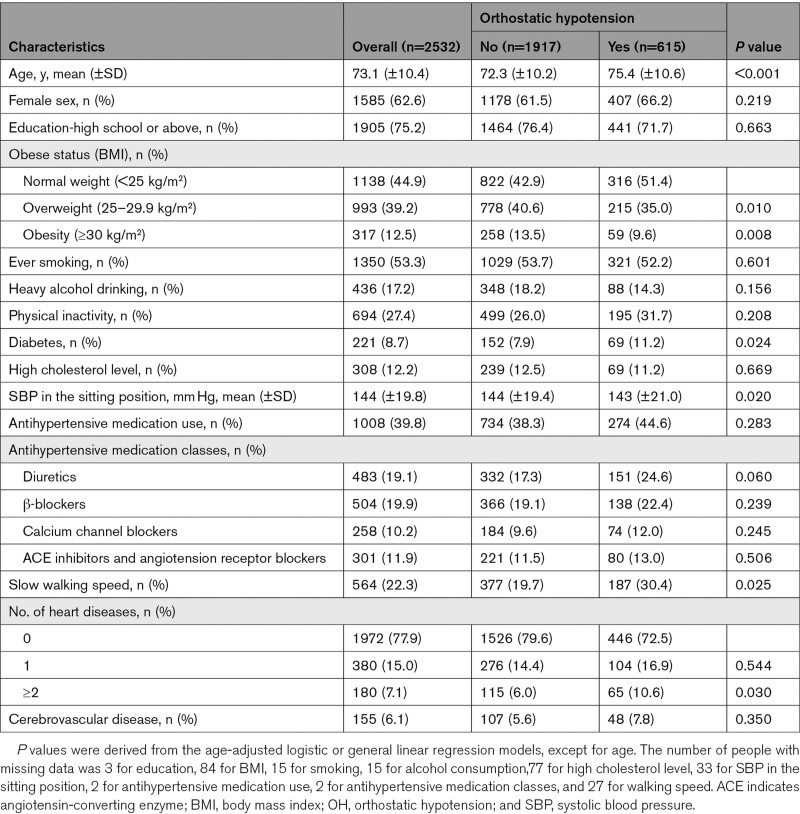
Baseline Characteristics of the Study Participants by OH Status in the Dementia-Free Cohort

**Figure 2.**
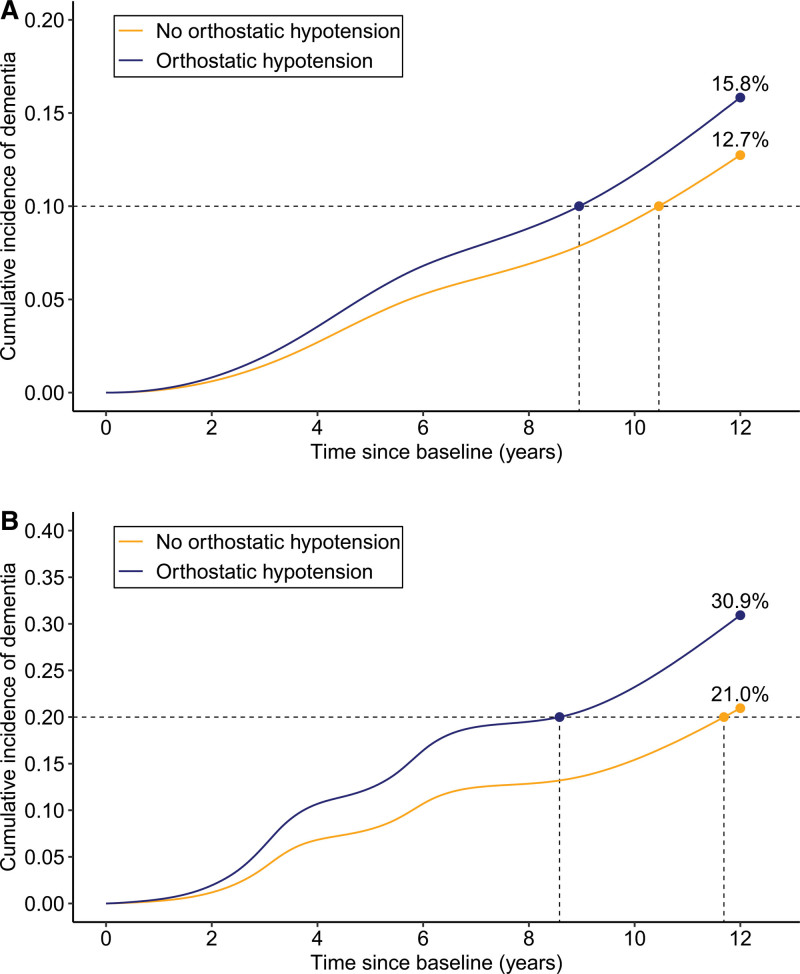
**Cumulative incidence of dementia by orthostatic hypotension (OH) status.A**, In the dementia-free cohort (n=2532). **B**, In the cognitive impairment, no dementia (CIND) cohort (n=576). Results were from flexible parametric models taking competing risk of death into account, adjusting for age, sex, education, body mass index, smoking, heavy alcohol drinking, physical inactivity, diabetes, high cholesterol level, systolic blood pressure in the sitting position, antihypertensive medication use, slow walking speed, number of heart diseases, and cerebrovascular disease.

During the 12-year follow-up period (mean, 8.5 years; SD, 3.7 years), 322 (12.7%) participants developed dementia, including 211 (8.3%) with AD. Controlling for demographic factors, OH was significantly associated with an increased risk of dementia and AD, and the associations remained similar after further controlling for vascular risk factors, motor function, and comorbid heart diseases and cerebrovascular disease (Table 2). Severe OH was more strongly associated with dementia and AD compared with mild OH (Table 2). The time by which 10% of people with OH developed dementia was ≈9 years since baseline and 10.5 years for those without OH (Figure 2A).

**Table 2.**
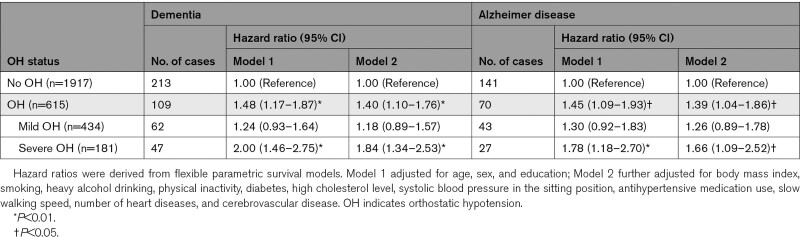
Associations of Baseline OH With Incident Dementia and Alzheimer Disease

Of the 1535 CIND-free participants at baseline, 331 (21.6%) were detected with OH (297 without and 34 with symptoms). During a mean follow-up period of 7.9 years (SD, 3.5 years), 546 (35.6%) developed CIND. OH was associated with an increased risk of CIND, but the association was not significant (Table 3).

**Table 3.**
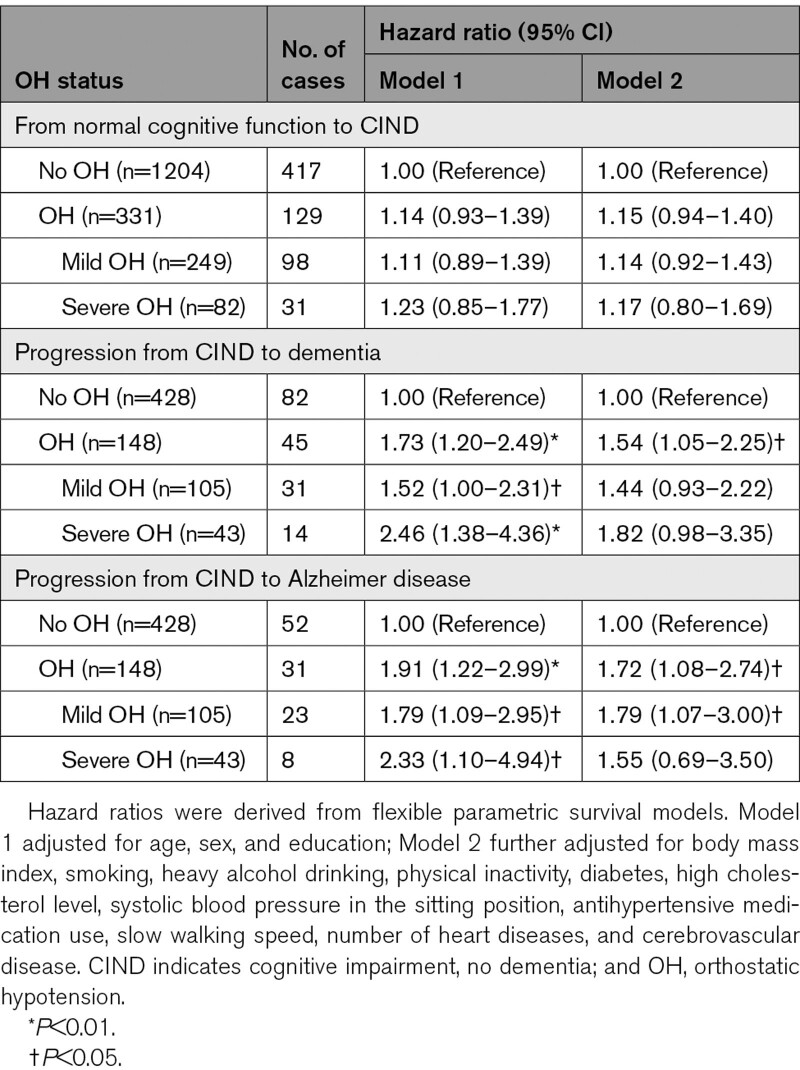
Associations of Baseline OH With Incident CIND and CIND Progression to Dementia

In the 576 individuals with CIND at baseline, 148 (25.7%) had OH (127 without and 21 with symptoms; 105 mild OH and 43 severe OH), and 127 (22.0%) developed dementia (83 with AD) during a mean follow-up period of 7.6 years (SD 3.9 years). OH was significantly associated with an elevated risk of CIND progression to dementia and AD, while only mild OH was associated with an increased risk of CIND progression to AD (Table 3). The time by which 20% of individuals developed dementia was 8.6 years in people with OH and 11.7 years in those without OH (Figure 2B). We did not find statistical interaction of OH with sex or *APOE* ε4 allele in the association of OH with dementia, CIND, or CIND progression to dementia.

**Figure 3.**
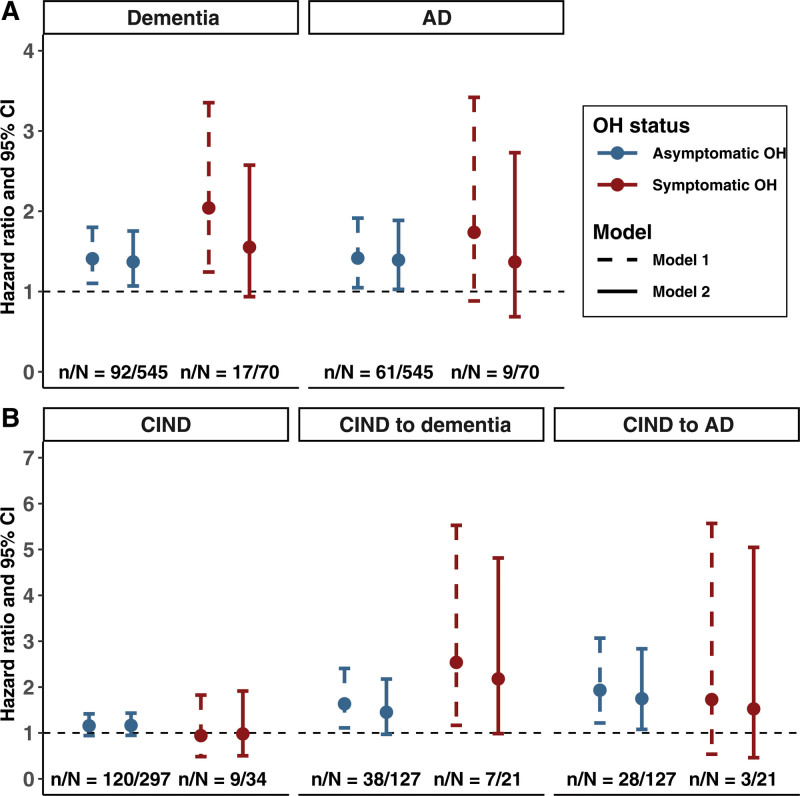
**The associations of asymptomatic and symptomatic orthostatic hypotension (OH) with cognitive outcomes, compared with people without OH.A**, The associations of OH with dementia (n=2532). **B**, The associations of OH with cognitive impairment, no dementia (CIND; n=1535) and CIND progression to dementia (n=576). Results were derived from flexible parametric survival models, and n/N indicates the number of outcome events/the number of participants. Model 1 adjusted for age, sex, and education; model 2 further adjusted for body mass index, smoking, heavy alcohol drinking, physical inactivity, diabetes, high cholesterol level, systolic blood pressure in the sitting position, antihypertensive medication use, slow walking speed, number of heart diseases, and cerebrovascular disease. AD indicates Alzheimer disease.

Further analysis by the presence of OH-related symptoms suggested that symptomatic OH had a stronger association with dementia than asymptomatic OH; however, the association with symptomatic OH was no longer significant when adjusted for all confounding factors (Figure 3A). In addition, neither symptomatic nor asymptomatic OH at baseline was significantly associated with incident CIND (Figure 3B). Finally, symptomatic OH was associated with the progression from CIND to dementia (Figure 3B).

The sensitivity analyses showed that when the more stringent definition was applied to defining OH for people with supine hypertension, the associations of OH with dementia, AD, and CIND progression to dementia and AD became stronger; besides, symptomatic OH was significantly associated with an elevated risk of dementia and CIND progression to dementia (Table S1 in the Data Supplement). Furthermore, when dividing OH according to possible etiology, the results showed that both non-neurogenic and neurogenic OH were significantly associated with an increased risk of dementia, whereas only non-neurogenic OH was significantly associated with CIND progression to dementia and AD (Table S2). Finally, after excluding 282 (11.1%) people with follow-up time shorter than 3 years, OH was still significantly associated with dementia and AD (Table S3).

## Discussion

This population-based cohort study of older adults indicates that OH, even asymptomatic OH, is associated with an elevated risk of dementia and CIND progression to dementia. Demographic factors, motor function, and cardiovascular burdens could not fully explain these associations. In addition, reverse causality may not fully account for the association of OH with dementia.

Several population-based longitudinal studies of middle-aged and older cohorts have shown OH was associated with an increased risk for dementia.^2–6^ For instance, the ARIC study (Atherosclerosis Risk in Communities) of middle-aged adults (mean age 54 years) in the United States found that baseline OH was associated with a 54% increased risk of dementia over a 24-year follow-up period.^5^ In contrast, the Malmö Preventive Project (mean age 45 years) in southern Sweden did not suggest such an association, possibly due to the low prevalence of OH in middle-aged adults and an underdiagnosis of dementia cases.^31^ Population-based studies of older ages generally supported a weak-to-moderate association between OH and the risk of dementia.^2–4,6^ The 12-year follow-up data from the Three-City study in people aged over 65 years showed that OH was related to a 24% increased risk of dementia^3^; the 25-year follow-up data from the Rotterdam study (mean age 68.5 years) showed that OH was associated with a 15% increased risk of dementia.^4^ The finding of the stronger association of dementia with severe OH than with mild OH was also in line with the Three-City study.^3^ Neither of the Three-City study and the Rotterdam study showed an association of OH with AD.^3,4^ In contrast, we found that OH was associated with an elevated risk not only for dementia but also for AD. However, people clinically diagnosed with AD are often accompanied by cerebrovascular pathology,^32–34^ which should be kept in mind when interpreting the findings of the associations of OH with AD and CIND progression to AD.

Evidence for the association between OH and cognitive impairment is inconclusive. The SNAC-Skåne study in southern Sweden did not find an association between OH and incident mild cognitive impairment.^2^ In contrast, the Amsterdam Dementia Cohort study of the memory clinic setting found that OH was associated with an increased risk of incident mild cognitive impairment or dementia.^19^ Furthermore, previous studies on the association between OH and cognitive decline showed mixed results.^5,11–18^ Data from the HYVET cohort (Hypertension in the Very Elderly Trial cohort) suggested OH was associated with increased risk of cognitive decline over 2 years of follow-up^16^; the TILDA (Irish Longitudinal Study on Ageing) suggested that OH was associated with global cognitive decline, mainly driven by the decline in executive function.^17^ Besides, data from the Tübingen Risk Evaluation for Neurodegenerative Diseases study of people aged 50 to 80 years in Germany showed that people with OH experienced a faster decline in global cognition, executive function, and memory.^18^ However, data from the ARIC study and a few population-based studies of older adults did not support an association between OH and cognitive decline.^5,11–15^ The lack of association between OH and mild cognitive impairment or cognitive decline in these studies could be partly due to the short follow-up time and the use of the Mini-Mental State Examination in the assessment of cognitive function,^2,11,12,15,35^ which has low sensitivity to detect changes in certain cognitive domains such as executive function. Our study had a relatively long follow-up period and used a comprehensive neuropsychological battery to define cognitive impairment but still did not find a significant association between OH and CIND. However, the missingness in CIND during the follow-up period might lead to underestimation of the association in our study. Therefore, the OH-CIND association deserves further investigation.

Orthostatic symptoms can be a sign of both OH severity and poor cerebral autoregulation,^7–9^ thus having important clinical implications. However, few studies on the associations of OH with cognitive impairment and dementia have considered orthostatic symptoms.^2,16^ The SNAC-Skåne study showed symptomatic subclinical OH (ie, the BP decrease does not meet the OH definition) but not OH was associated with mild cognitive impairment.^2^ The HYVET study demonstrated a stronger association between symptomatic subclinical OH and dementia compared with OH.^16^ Our study did not find associations of symptomatic OH with CIND or dementia partly owing to limited statistical power, but the results did suggest that symptomatic OH was associated with an increased risk of CIND progression to dementia. Further large-scale studies are warranted to clarify the role of orthostatic symptoms in the relationship between OH and cognitive impairment and dementia. Meanwhile, our results showed that asymptomatic OH was associated with dementia and CIND progression to dementia, suggesting that asymptomatic OH may deserve attention in clinical practice.

Cerebral hypoperfusion induced by OH could explain the associations of OH with adverse cognitive outcomes. We previously reported that low BP in very old age was associated with a higher risk of dementia and AD.^36^ A plausible explanation is that low BP results in insufficient cerebral blood flow, which may increase the risk of dementia.^37^ In the current study, the associations of OH with dementia and CIND progression remained evident after adjusting for the sitting SBP, suggesting that the effect of OH on cognition was independent of low sitting BP. Postural changes from a supine to a standing position can lead to a redistribution of blood to lower extremities and a reduction in BP, which may also result in reduced cerebral blood flow, and repeated episodes of OH may be responsible for cerebral ischemic damage and an elevated risk for dementia.^1,22,37^ People who experience orthostatic symptoms are more likely to have insufficient cerebral blood perfusion,^9^ which may partly explain the slightly stronger association of symptomatic OH with the progression from CIND to dementia than that of asymptomatic OH. The association of OH with CIND was not evident in our study. Similarly, previous studies demonstrated that OH was related to an elevated risk for dementia but not faster cognitive decline or cognitive impairment.^2,5^ These findings suggest that OH may be more harmful to people that already have cognitive impairment. Alternatively, OH can be an early sign of underlying neuropathological changes in the brain. BP responses to postural changes are regulated by baroreflex in coordination with the central autonomic network, which involves multiple brain regions (eg, hypothalamus, insular cortex, and amygdala).^1^ The regulation could be altered in the context of neurodegenerative conditions.^38–40^ However, our data showed that both non-neurogenic and neurogenic OH were associated with dementia and CIND progression and that the association of OH with dementia remained evident even when introducing a lag period of 3 years between OH assessment and dementia diagnosis. These findings imply that the underlying neurodegeneration is unlikely to fully account for the associations of OH with cognitive outcomes in older adults.

This study has limitations. First, supine and standing BP was only measured once, which may affect the accuracy of the assessments and lead to nondifferential misclassification of exposure and underestimation of the true association. Second, we were unable to examine the effect of delayed OH (ie, OH occurs after 3 minutes of active standing) owing to lacking relevant data, which may have a more profound impact on cognition.^17,19^ Third, although we have controlled for a broad range of potential confounding factors, we cannot completely rule out residual confounding bias due to imperfect measurements of some factors and unmeasured confounders. Fourth, we have limited power to detect the weak-to-moderate associations of symptomatic OH with cognitive outcomes due to the relatively few people with symptomatic OH. Finally, the study population has a better socioeconomic status compared with other regions of Sweden. Therefore, caution should be taken when generalizing our research findings.

In conclusion, this population-based cohort study suggests that OH, even asymptomatic OH, is associated with an increased risk of dementia and the progression from CIND to dementia in older adults. Further studies with beat-to-beat BP measures, neuropathological biomarkers, and vascular aging measures could help clarify the mechanisms underlying these associations. In addition, the early identification of OH in older adults may help identify individuals at risk for dementia and implement timely actions to address the underlying causes of OH and thereafter prevent or delay dementia onset. This is possible given that OH can be managed by nonpharmacological and pharmacological interventions.

## Perspectives

OH is a common and usually asymptomatic condition in older adults and associated with an elevated risk for dementia and accelerated progression from CIND to dementia. However, further investigations are warranted to clarify the mechanisms underlying these associations.

## Acknowledgments

We thank all the SNAC-K (Swedish National Study on Aging and Care in Kungsholmen) participants and the SNAC-K group for their collaboration in data collection and management.

## Sources of Funding

The SNAC-K (Swedish National Study on Aging and Care in Kungsholmen) is supported by the Swedish Ministry of Health and Social Affairs and the participating county councils and municipalities, and in part by additional grants from the Swedish Research Council (2017-06088) and the Swedish Research Council for Health, Working Life and Welfare (2016-07175).

## Disclosures

X. Xia was supported, in part, by a scholarship from the China Scholarship Council (201907930019) and by a grant from the National Graduate School on Ageing and Health (SWEAH). R. Wang received a grant from the Swedish Research Council (2016-06658). C. Qiu received grants from the Swedish Research Council (2017-00740, 2017-05819, and 2020-01574), the Swedish Foundation for International Cooperation in Research and Higher Education (CH2019-8320), and Karolinska Institutet, Stockholm, Sweden. The other authors report no conflicts.

## Supplementary Material



## Novelty and Significance

### What Is New?

- The study suggests that orthostatic hypotension, even when asymptomatic, is associated with worse cognitive outcomes in older adults.

### What Is Relevant?

- Short-term blood pressure instability, which can be induced by postural change, may play a role in accelerating cognitive aging.

### Summary

Our study found that orthostatic hypotension, even asymptomatic orthostatic hypotension, was associated with an increased risk of dementia and accelerated progression from cognitive impairment to dementia. Further research is needed to understand the mechanisms underlying these associations.
