# Supplementary material for: From Normal Cognition to Cognitive Impairment and Dementia: Impact of Orthostatic Hypotension
Source: Hypertension. 2021 Jul 6;78(3):769–78. doi: 10.1161/HYPERTENSIONAHA.121.17454 (PMC8357050; doi:10.1161/HYPERTENSIONAHA.121.17454)
Supplement: Supplementary file 1 [file hyp-78-769-s001.pdf]

DATA SUPPLEMENTS

Full title: From normal cognition to cognitive impairment and dementia: impact of orthostatic hypotension

Short title: Orthostatic Hypotension and Cognitive Aging

Authors: Xin Xia MSc[1], Rui Wang PhD[1,2,3], Davide L. Vetrano MD, PhD[1,4,5], Giulia Grande MD, PhD[1], Erika J. Laukka PhD[1,6], Mozhu Ding PhD[1,7], Laura Fratiglioni MD, PhD[1,6], Chengxuan Qiu PhD[1*]

Affiliations:

[1]Aging Research Center, Department of Neurobiology, Care Sciences and Society (NVS), Karolinska Institutet-Stockholm University, Stockholm, Sweden
[2]The Swedish School of Sport and Health Sciences, GIH, Stockholm, Sweden
[3]Department of Medicine and Wisconsin Alzheimer's Disease Research Center, University of Wisconsin School of Medicine and Public Health, Madison, WI, USA
[4]Department of Geriatrics, Catholic University of Rome, Rome, Italy
[5]Centro di Medicina dell'Invecchiamento, Fondazione Policlinico A. Gemelli, Rome, Italy
[6]Stockholm Gerontology Research Center, Stockholm, Sweden
[7]Unit of Epidemiology, Institute of Environmental Medicine, Karolinska Institutet, Stockholm, Sweden

Correspondence: Dr Chengxuan Qiu, Aging Research Center, Karolinska Institutet, Widerströmska Huset, Tomtebodavägen 18A, 171 65 Solna, Sweden. Tel.: +46 852485821, Email: chengxuan.qiu@ki.se

Supplemental Methods:

Definition of OH by supine hypertension status
OH was defined as a ≥20/10 mmHg decline in SBP/DBP when standing up from the supine position for people without supine hypertension. For people with supine hypertension (SBP/DBP ≥150/90 mmHg in the supine position), OH was defined as a ≥30/15 mmHg decline in SBP/DBP.[1]

Definition of neurogenic OH
Neurogenic OH was defined among people with OH as the ratio of the heart rate increase to SBP decline <0.5 bpm/mmHg when standing up from the supine position.[2]

Supplemental References:

1. Gibbons CH, Schmidt P, Biaggioni I, Frazier-Mills C, Freeman R, Isaacson S, Karabin B, Kuritzky L, Lew M, Low P, et al. The recommendations of a consensus panel for the screening, diagnosis, and treatment of neurogenic orthostatic hypotension and associated supine hypertension. J Neurol. 2017;264(8):1567-1582. doi: 10.1007/s00415-016-8375-x.

2. Norcliffe-Kaufmann L, Kaufmann H, Palma JA, Shibao CA, Biaggioni I, Peltier AC, Singer W, Low PA, Goldstein DS, Gibbons CH, et al; Autonomic Disorders Consortium. Orthostatic heart rate changes in patients with autonomic failure caused by neurodegenerative synucleinopathies. Ann Neurol. 2018;83(3):522-531. doi: 10.1002/ana.25170.

Supplemental Tables

Table S1. Associations of baseline orthostatic hypotension with cognitive outcomes

**OH status**	**No. of cases**	**Hazard ratio (95% CI)**	
		**Model 1**	**Model 2**
**Dementia**			
**No OH (n = 2145)**	244	1.00 (Reference)	1.00 (Reference)
**OH (n = 386)**	77	1.91 (1.48-2.47)*	1.72 (1.32-2.24)*
**Asymptomatic OH (n = 333)**	64	1.80 (1.36-2.37)*	1.65 (1.25-2.19)*
**Symptomatic OH (n = 53)**	13	2.77 (1.58-4.85)*	2.21 (1.25-3.90)*
**Alzheimer's disease**			
**No OH (n = 2145)**	160	1.00 (Reference)	1.00 (Reference)
**OH (n = 386)**	51	1.96 (1.43-2.70)*	1.81 (1.31-2.50)*
**Asymptomatic OH (n = 333)**	44	1.91 (1.37-2.67)*	1.79 (1.27-2.52)*
**Symptomatic OH (n = 53)**	7	2.38 (1.11-5.09)†	1.95 (0.90-4.22)
**From normal cognitive function to CIND**			
**No OH (n = 1334)**	476	1.00 (Reference)	1.00 (Reference)
**OH (n = 201)**	70	1.08 (0.84-1.39)	1.05 (0.81-1.36)
**Asymptomatic OH (n = 176)**	64	1.12 (0.86-1.45)	1.08 (0.83-1.41)
**Symptomatic OH (n = 25)**	6	0.79 (0.35-1.77)	0.83 (0.36-1.87)
**Progression from CIND to dementia**			
**No OH (n = 483)**	94	1.00 (Reference)	1.00 (Reference)
**OH (n = 93)**	33	2.38 (1.59-3.56)*	1.78 (1.14-2.80)†
**Asymptomatic OH (n = 75)**	27	2.17 (1.41-3.34)*	1.63 (1.02-2.63)†
**Symptomatic OH (n = 18)**	6	4.31 (1.86-9.94)*	3.20 (1.32-7.71)†
**Progression from CIND to Alzheimer's disease**			
**No OH (n = 483)**	61	1.00 (Reference)	1.00 (Reference)
**OH (n = 93)**	22	2.74 (1.67-4.51)*	2.04 (1.18-3.55)†
**Asymptomatic OH (n = 75)**	19	2.64	1.97

		(1.56-4.46)*	(1.10-3.51)†
**Symptomatic OH (n = 18)**	3	3.59 (1.12-11.55)†	2.77 (0.80-9.54)

Hazard ratios (95% confidence interval) were derived from flexible parametric survival models. Model 1 adjusted for age, sex, and education; Model 2 adjusted for age, sex, education, body mass index, smoking, heavy alcohol drinking, physical inactivity, diabetes, high cholesterol level, systolic blood pressure in the sitting position, antihypertensive medication use, slow walking speed, number of heart diseases, and cerebrovascular disease. OH indicates orthostatic hypotension; and CIND, cognitive impairment, no dementia.
*$P<0.01$.
†$P<0.05$.

Table S2. Associations of baseline non-neurogenic and neurogenic orthostatic hypotension with cognitive outcomes

OH status	No. of cases	Hazard ratio (95% CI)	
		Model 1	Model 2
**Dementia**			
**No OH (n = 1917)**	213	1.00 (Reference)	1.00 (Reference)
**Non-neurogenic OH (n = 253)**	42	1.53 (1.10-2.13)†	1.51 (1.08-2.11)†
**Neurogenic OH (n = 345)**	65	1.45 (1.10-1.92)*	1.35 (1.02-1.79)†
**Alzheimer's disease**			
**No OH (n = 1917)**	141	1.00 (Reference)	1.00 (Reference)
**Non-neurogenic OH (n = 253)**	27	1.51 (1.00-2.28)†	1.51 (1.00-2.29)
**Neurogenic OH (n = 345)**	42	1.43 (1.01-2.02)†	1.34 (0.94-1.90)
**From normal cognitive function to CIND**			
**No OH (n = 1204)**	417	1.00 (Reference)	1.00 (Reference)
**Non-neurogenic OH (n = 139)**	48	1.07 (0.80-1.45)	1.03 (0.76-1.40)
**Neurogenic OH (n = 185)**	78	1.18 (0.93-1.50)	1.23 (0.97-1.58)
**Progression from CIND to dementia**			
**No OH (n = 428)**	82	1.00 (Reference)	1.00 (Reference)
**Non-neurogenic OH (n = 61)**	21	2.00 (1.24-3.25)*	2.02 (1.22-3.35)*
**Neurogenic OH (n = 78)**	22	1.54 (0.96-2.47)	1.30 (0.79-2.13)
**Progression from CIND to Alzheimer's disease**			
**No OH (n = 428)**	52	1.00 (Reference)	1.00 (Reference)
**Non-neurogenic OH (n = 61)**	15	2.21 (1.24-3.93)*	2.44 (1.34-4.45)*
**Neurogenic OH (n = 78)**	15	1.71 (0.96-3.04)	1.37 (0.74-2.51)

Hazard ratios (95% confidence interval) were derived from flexible parametric survival models. Model 1 adjusted for age, sex, and education; Model 2 adjusted for age, sex, education, body mass index, smoking, heavy alcohol drinking, physical inactivity, diabetes, high cholesterol level, systolic blood pressure in the sitting position, antihypertensive medication use, slow walking speed, number of heart diseases, and cerebrovascular disease. OH indicates orthostatic hypotension; and CIND, cognitive impairment, no dementia.

*P<0.01.

†P<0.05.

Table S3. Associations of baseline orthostatic hypotension with incident dementia and Alzheimer's disease with a lag period of three years

OH status	Dementia			Alzheimer's disease		
	No. of cases	Hazard ratio (95% CI)		No. of cases	Hazard ratio (95% CI)	
		Model 1	Model 2		Model 1	Model 2
**No OH (n=1732)**	175	1.00 (Reference)	1.00 (Reference)	124	1.00 (Reference)	1.00 (Reference)
**OH (n=518)**	84	1.45 (1.12-1.89)*	1.40 (1.07-1.82)†	60	1.46 (1.07-1.98)†	1.39 (1.01-1.89)†
**Asymptomatic OH (n = 465)**	70	1.36 (1.03-1.80)†	1.35 (1.02-1.78)†	51	1.38 (1.00-1.92)	1.35 (0.97-1.88)
**Symptomatic OH (n= 53)**	14	2.21 (1.27-3.81)*	1.71 (0.98-2.98)	9	2.10 (1.06-4.15)†	1.65 (0.82-3.30)

Hazard ratios (95% confidence interval) were derived from flexible parametric survival models. Model 1 adjusted for age, sex, and education; Model 2 adjusted for age, sex, education, body mass index, smoking, heavy alcohol drinking, physical inactivity, diabetes, high cholesterol level, systolic blood pressure in the sitting position, antihypertensive medication use, slow walking speed, number of heart diseases, and cerebrovascular disease. OH indicates orthostatic hypotension.

*P<0.01.

†P<0.05.
