# Supplementary figures and images for: From Normal Cognition to Cognitive Impairment and Dementia: Impact of Orthostatic Hypotension
Source: Hypertension. 2021 Jul 6;78(3):769–78. doi: 10.1161/HYPERTENSIONAHA.121.17454 (PMC8357050; doi:10.1161/HYPERTENSIONAHA.121.17454)

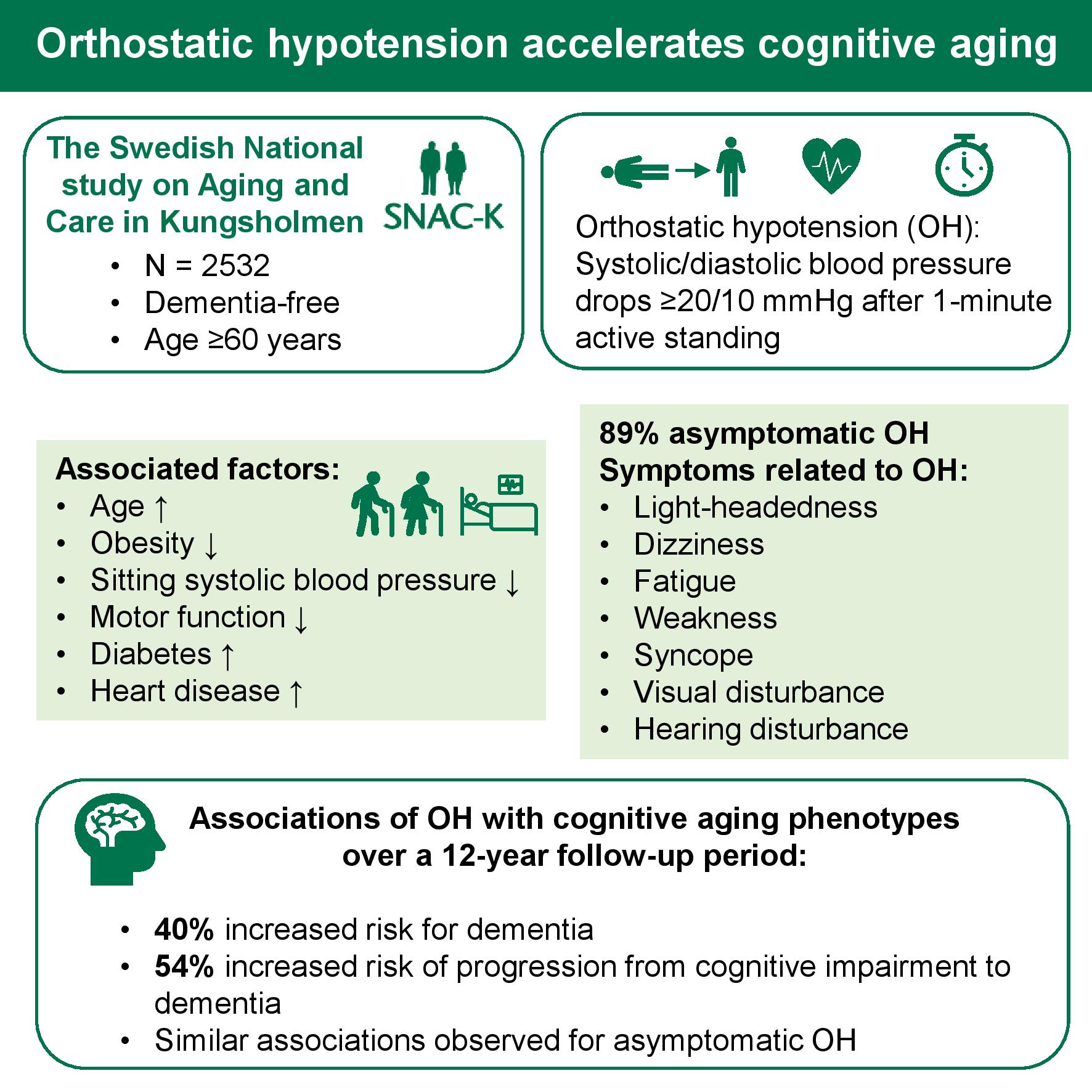

Supplement: Supplementary file 2 [file hyp-78-769-s002.jpg]
